# One Is Enough: *In Vivo* Effective Population Size Is Dose-Dependent for a Plant RNA Virus

**DOI:** 10.1371/journal.ppat.1002122

**Published:** 2011-07-07

**Authors:** Mark P. Zwart, José-Antonio Daròs, Santiago F. Elena

**Affiliations:** 1 Instituto de Biología Molecular y Celular de Plantas, Consejo Superior de Investigaciones Científicas-UPV, València, Spain; 2 The Santa Fe Institute, Santa Fe, New Mexico, United States of America; University of Kentucky, United States of America

## Abstract

Effective population size (*N_e_*) determines the strength of genetic drift and the frequency of co-infection by multiple genotypes, making it a key factor in viral evolution. Experimental estimates of *N_e_* for different plant viruses have, however, rendered diverging results. The independent action hypothesis (IAH) states that each virion has a probability of infection, and that virions act independent of one another during the infection process. A corollary of IAH is that *N_e_* must be dose dependent. A test of IAH for a plant virus has not been reported yet. Here we perform a test of an IAH infection model using a plant RNA virus, *Tobacco etch virus* (TEV) variants carrying GFP or mCherry fluorescent markers, in *Nicotiana tabacum* and *Capsicum annuum* plants. The number of primary infection foci increased linearly with dose, and was similar to a Poisson distribution. At high doses, primary infection foci containing both genotypes were found at a low frequency (<2%). The probability that a genotype that infected the inoculated leaf would systemically infect that plant was near 1, although in a few rare cases genotypes could be trapped in the inoculated leaf by being physically surrounded by the other genotype. The frequency of mixed-genotype infection could be predicted from the mean number of primary infection foci using the independent-action model. Independent action appears to hold for TEV, and *N_e_* is therefore dose-dependent for this plant RNA virus. The mean number of virions causing systemic infection can be very small, and approaches 1 at low doses. Dose-dependency in TEV suggests that comparison of *N_e_* estimates for different viruses are not very meaningful unless dose effects are taken into consideration.

## Author Summary

Plant viruses have been used to test quantitative models of infection for over 80 years. Recent advances in molecular biology have made it possible to make empirical estimates of the effective population size, the number of virions (i.e., virus particles) that are actually infecting a plant. The relationship between effective population size and virion dose has not been elucidated, and there are discrepancies between estimates of effective population size made for different plant viruses. We therefore formulated a simple model of infection based on the independent action hypothesis, the idea that each virion can infect the plant independently of the presence of other virions. Next, we exposed plants to different doses of two viral genotypes carrying fluorescent markers, which allow for tracking infection. We found that our infection model could describe the overall rate of infection, the number of starting infections, and the number of virus genotypes infecting the entire plant. These tests show that effective population size is dependent on dose and proportional to the number of virions the plant has been exposed to. The minimum effective population size is 1, suggesting that genetic drift can play a dominant role in the evolution of plant viruses.

## Introduction

Viral evolutionary dynamics on different spatiotemporal scales will be largely determined and constrained by effective population size (*N_e_*). For viruses, we define *N_e_* as the average number of horizontally transmitted virions (i.e., reproducing individuals) in an idealized population [1], [2]. Here we focus exclusively on *N_e_* at the level of an individual host organism, although it could also be considered at the metapopulation level (i.e., a population of infected hosts). *N_e_* will determine how strong genetic drift – one of the key evolutionary forces – acts on viral populations [3]. Genetic drift can lead to decreases in fitness [4], [5] and virulence [6]. *N_e_* will also be an important determinant of mixed-genotype infections, in collusion with the standing genetic variation within a population [7]. The number of mixed-genotype infections will in turn determine the extent to which recombination [8], complementation [9], and competition [10], [11] can occur. Moreover, as it can constrain within-host competition, *N_e_* will also be important for determining the importance of different levels of selection [12], [13]. Estimates of *N_e_* are therefore indispensable for understanding viral evolution.

Estimates of viral *N_e_* would be simplified by understanding basic virus-host and virus-virus interactions during infection. The independent-action hypothesis (IAH) of micro-parasite infection [7], [14], [15], [16], [17] proposes that each virion has a non-zero probability of infection (virus-host interaction), and that the action of micro-parasite infectious entities (i.e., virions) is independent of the presence of other infectious entities (virus-virus interaction). We could therefore assign each virion a probability of infecting the host, which is fixed irrespective of the presence of other co-inoculated virions. The IAH model was first formulated for plant viruses [17], but has not been re-examined with more modern techniques in the past decades (reviewed in [18]). The model was used to better understand the number of local lesions in inoculated leaves [17], but the model is versatile and can be applied to systemic infection [7], [14] or other situations. For example, it has been shown that potyvirus inoculation by aphids is independent; the number of aphids the plant is exposed to does not affect the probability of infection per aphid [19]. Two important limitations of the IAH model are that it is strictly limited to: (i) challenge of the host at a single time point, and (ii) the virus-virus interactions between conspecific infectious entities. Dependency in infection probability between virions can occur. For example, synergistic interactions between virions have been reported for plant viruses [20], [21] and baculoviruses [22], [23], and cross-protection between plant viruses is a well-known antagonistic interaction [24]. However, these phenomena concern interactions between different genotypes, super-infection or both. The IAH model, on the other hand, considers only interactions between synchronously inoculated, conspecific virions.

If virus infection is characterized by independent action, *N_e_* is simply the product of infection probability and the number of virions the host is challenged with. Under the IAH model, it would therefore be possible to predict *N_e_* at multiple doses. Moreover, when IAH holds, viral population size is inextricably linked to the level of host infection and can be estimated directly from the rate of host infection [7]. However, no studies have addressed whether the IAH model pertains to the infection process of RNA viruses or plant viruses. Independent action has only been reported for an animal DNA virus, and was only applicable in two out of six pathosystems tested [7]. The generality of the independent-action model is therefore questionable.

Plant RNA viruses have been important model systems for empirical estimates of *N_e_*. Moury *et al.* found an *N_e_* of 0.5–3.2 individuals when considering transmission of *Potato virus Y* (PVY) between plants by aphids [25]. Ali *et al.* found that aphid transmission significantly reduced the genetic variation in an experimental *Cucumber mosaic virus* (CMV) population. Moreover, they identified that the reduction occurred not during acquisition of the virus by aphids, but rather during the infection of new plants [26]. Although estimates of the number of individuals were not made, 1–7 genotypes were recovered from infected plants. This suggests a small population size, but not on the order of magnitude of units. Finally, Betancourt *et al.* performed similar experiments with CMV, and estimated a population size of one to two individuals [27]. Low estimates of *N_e_* appear to be the norm for transmission experiments.

Estimates of *N_e_* have also been made when considering bottlenecks imposed by systemic colonization. Hall *et al.* found evidence for colonization of tillers by very small populations of *Wheat streak mosaic virus* (WSMV) [28], estimated at a size of four individuals [29]. Sacristan *et al.* found a similar result for the systemic colonization of plants by *Tobacco mosaic virus* (TMV) [30]. For CMV, a reanalysis of the data gathered by Li and Roossinck [31] lead to slightly larger population size estimates of 12–220 individuals [32]. More recently, Monsion *et al.* estimated that *N_e_* for the pararetrovirus *Cauliflower mosaic virus* (CaMV), a dsDNA virus, was approx. 300–500 individuals [33]. Whereas studies with different viruses have somewhat similar estimates for *N_e_* during horizontal transmission, there are discrepancies between estimates of *N_e_* for systemic colonization. Although differences in experimental setup will undoubtedly contribute to the variation in estimates of *N_e_*, what other factors contribute?

Here we address two related emerging questions about the infection biology of plant RNA viruses. First, can the infection process of a plant RNA virus be characterized by independent action? Second, could the variation in *N_e_* estimates be due in part to dose-dependence? We hypothesize that the IAH model can characterize the infection process of a mono-partite RNA plant virus. As a corollary, we also predict that *N_e_* will be dose-dependent. If *N_e_* is dose-dependent, we may have uncovered an important potential source of variation between studies performed on different viruses. To test these hypotheses, we constructed two *Tobacco etch virus* (TEV, genus *Potyvirus*, family *Potyviridae*) genotypes, which differed only in the color of the incorporated fluorescent marker. We then challenged highly susceptible tobacco plants (*Nicotiana tabacum*), and less susceptible pepper plants (*Capsicum annuum*), with different viral doses. We determined the number of primary infection foci in the inoculated leaf, and which genotypes had systemically infected the plant, and compared these data to predictions of a simple IAH model.

## Results/Discussion

### The number of primary infection foci in inoculated leaves is dose dependent

**Figure 1:**
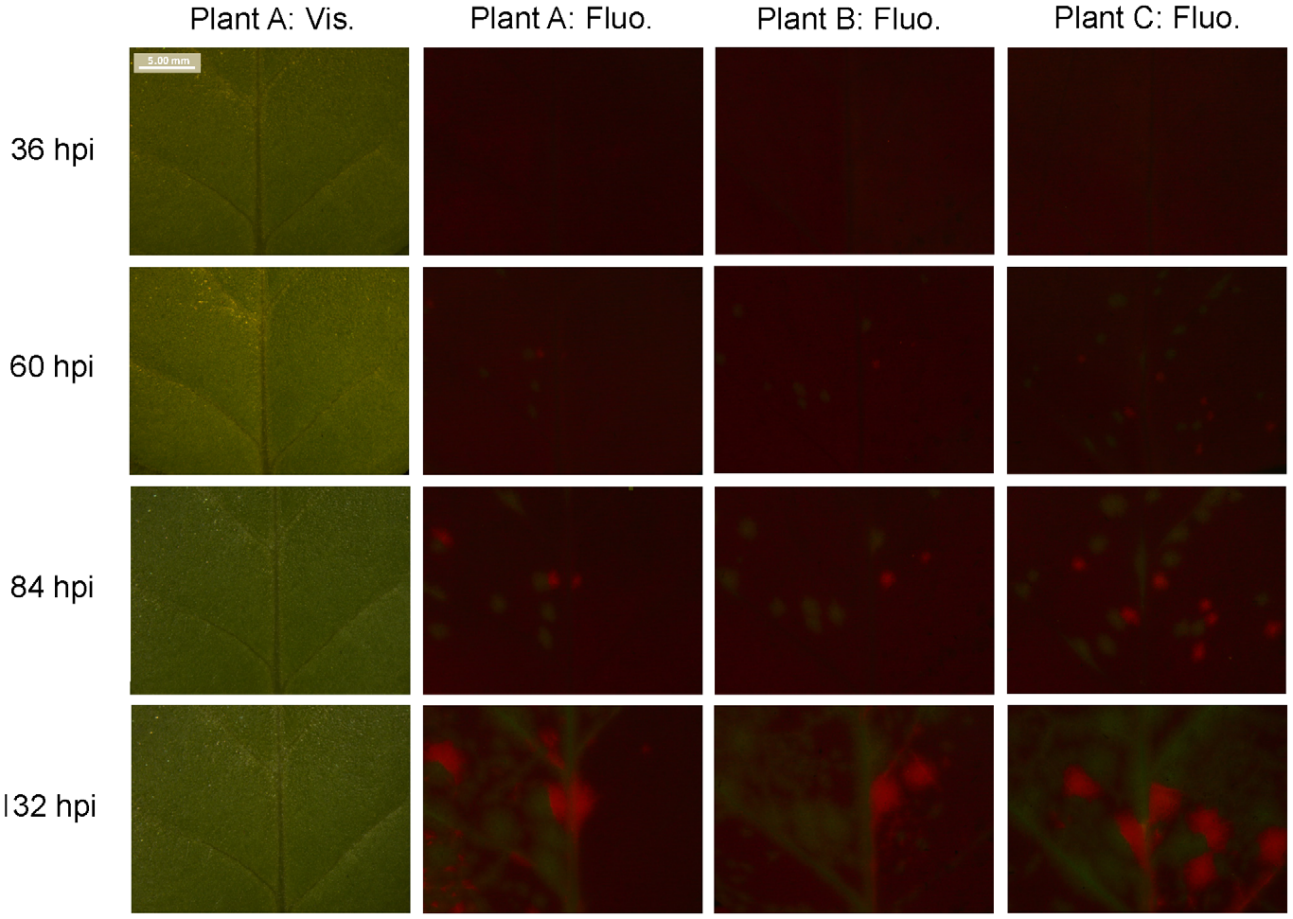
Fluorescence time course in inoculated *N. tabacum* leaves. The development of foci of primary infection in the inoculated leaf of *N. tabacum* was followed over time. Patterns in vascular tissue, observed under visible light through a stereomicroscope (‘Plant A: Vis.’), were used to track fluorescence in the same region. GFP and mCherry signals were merged, and given for three example plants (‘Plant A: Fluo.’ through ‘Plant C: Fluo.’). The best time point for viewing foci of primary infection is 84 hpi: the foci have reasonably high levels of fluorescence, but the fluorescence has not spread.

*N. tabacum* and *C. annuum* plants were inoculated with a three-fold dilution series of purified TEV virions. These virions were a 1∶1 mix of TEV-GFP, a TEV infectious clone [34], tagged with a GFP (green fluorescent protein) marker inserted between P1 and HC-Pro cistrons, and TEV-mCherry, tagged with mCherry marker (a red fluorescent reporter protein [35]) in the same location. We confirmed the stability of these constructs after three serial passages in *N. tabacum* plants (see Materials and Methods). The fluorescent reporter proteins allowed us to quantify the number of primary infection foci in inoculated leaves. A time-course experiment was performed to determine when the number of foci could be quantified best. The data indicated that fluorescence could be quantified best at 84 hours post inoculation (hpi), for both plant species. At this time, virus-induced fluorescence was high and had not visibly spread from the site of primary infection in both *N. tabacum* (Fig. 1) and *C. annuum* (Fig. S1; see Supplementary Materials).

**Figure 2:**
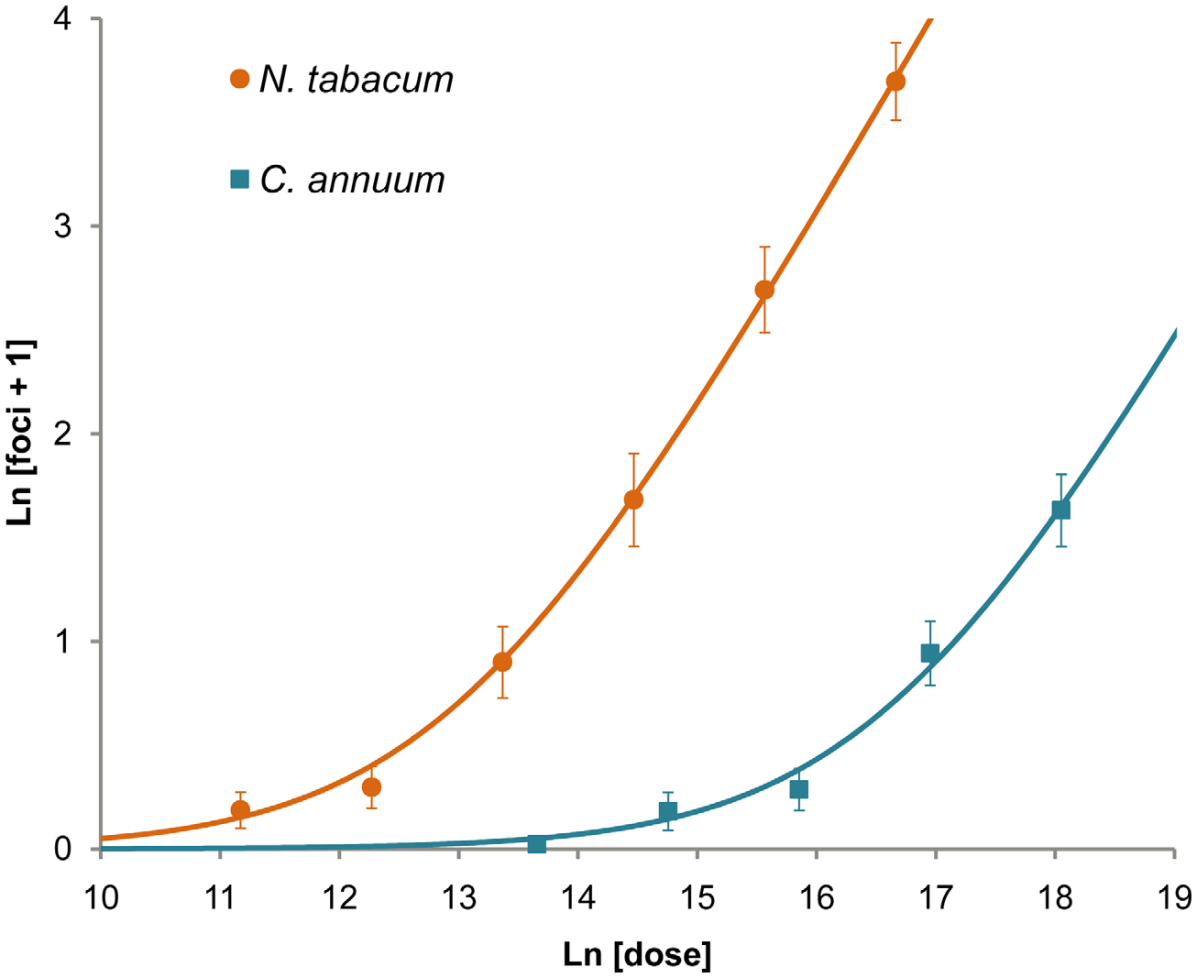
Number of primary infection foci in inoculated leaves at different doses. The ordinate is the mean ln-transformed number of foci of primary infection plus one, with error bars indicating 95% confidence intervals, while the abscise is ln-transformed dose (the number of virions per inoculated plant, as estimated by the number of RNA molecules measured by RT-qPCR). The orange circles and blue squares are data points for *N. tabacum* and *C. annuum*, respectively. The orange and blue lines are the fitted IAH dose-foci relationship. A good model fit for *N. tabacum* (*R* ^2^ = 0.713, *F*_1,21_ = 52.091, *P*<0.001) and *C. annuum* (*R* ^2^ = 0.799, *F*_1,12_ = 47.636, *P*<0.001) indicates number of foci increase linearly with dose. Note that the data and model taper off as the mean number of foci becomes low due to the ln transformation.

We then recorded the number of GFP and mCherry foci in the inoculated leaf (*λ*). Fifteen plants were used per replicate and dose, and four replicates were performed with *N. tabacum* and three with *C. annuum*. We fitted the expected dose-foci relationship (see Materials and Methods) and found a good fit of the IAH model for *N. tabacum* and *C. annuum* (Fig. 2), indicating that the number of foci increased linearly with dose, as would be expected under IAH. However, the slope of the relationship was different among the two hosts (ANCOVA, *F*_1,33_ = 70.385, *P*<0.001), being 36.4-times larger for *N. tabacum*. This result indicates that for a given increase in dose, the number of foci produced was disproportionately larger in *N. tabacum* leaves than in *C. annuum* leaves, thus confirming that tobacco is a much more susceptible host for TEV than pepper. We conclude that the number of foci of primary infection in the inoculated leaf is dose and host-species dependent, with a greater number of foci found at higher doses in the more susceptible host.

Our results are similar to those obtained when quantifying the number of local lesions for plant viruses [15], [16], [17], [36], [37], [38]. However, care should be taken when comparing primary infection foci and local lesions, as there may not be one-to-one correspondence. Moreover, others have reported that the number of local lesions is not proportional to dose for relatively high doses, the number of foci being less than predicted [15], [35], [38]. We did not use extremely high doses here (>100 foci per leaf), perhaps explaining why this effect was not seen.

### Number of foci follows a Poisson distribution

If virions act independently and each focus of primary infection is typically initiated by a single virion, a point addressed below, the mean number of foci of primary infection will increase linearly with dose. Furthermore, if the probability of infection is also constant over host individuals (i.e., no heterogeneity in susceptibility) then, for any given dose, the number of foci (i.e., the number of infecting micro-parasites) is expected to follow a Poisson distribution [7], [15], [16]. We therefore performed a one-sample Kolmogorov-Smirnoff test to determine whether the distribution of foci of primary infection follows a Poisson distribution. As there was a block effect on the number of foci at the highest dose for both *N. tabacum* (ANOVA, *F*_3,54_ = 41.512, *P*<0.001) and *C. annuum* (ANOVA, *F*_2,42_ = 6.968, *P* = 0.002), we chose to analyze replicates separately. For both *N. tabacum* (Table 1) and *C. annuum* (Table 2), the data appear similar to a Poisson distribution. Only in *N. tabacum* do discrepancies exist at the highest dose, although we suspect that a similar pattern may emerge in *C. annuum* with even higher virion concentrations in the inoculum. Others have reported a distribution of the number of local lesions with more variance than a Poisson distribution, and concluded that this was due to differences in probability of infection between primary infection sites [36], [38]. However, for our experimental data, there is no reason to reject a fixed probability of infection per virion.

**Table 1:** Variance in the mean number of foci of primary infection for *N. tabacum*.

Dilution	Virion dose	Replicate	Mean foci	Variance	*Z*	*P*
1∶10	1.73×10^7^	1	19.92	141.58	1.518	0.020
		2	29.73	225.92	1.882	0.025
		3	66.20	316.46	1.140	0.149
		4	75.60	315.26	0.727	0.666
1∶30	5.75×10^6^	1	7.13	16.41	0.484	0.973
		2	9.00	36.57	1.007	0.263
		3	28.00	61.57	0.801	0.543
		4	29.33	87.38	0.919	0.367
1∶90	1.92×10^6^	1	1.53	2.27	0.246	1
		2	3.20	7.74	0.617	0.841
		3	11.00	12.00	0.468	0.981
		4	9.53	8.70	0.418	0.995
1∶270	6.39×10^5^	1	0.60	0.83	0.198	1
		2	1.53	2.98	0.713	0.689
		3	3.67	4.95	0.208	1
		4	2.47	1.84	0.332	1
1∶810	2.13×10^5^	1	0.33	0.24	0.193	1
		2	0.40	0.69	0.244	1
		3	0.27	0.21	0.126	1
		4	0.87	0.55	0.337	1
1∶2430	7.10×10^4^	1	N.D.			
		2	0.13	0.27	0.227	1
		3	0.40	0.40	0.031	1
		4	0.33	0.24	0.193	1

Dilution is the dilution of the purified virions used to inoculate plants, and virion dose is the number of virions per inoculated plant, as estimated by the number of RNA molecules measured by RT-qPCR. Mean foci is the mean number of foci of primary infection, of both TEV-GFP and TEV-mCherry, in an inoculated leaf. Variance is the calculated variance of the mean number of foci of primary infection. Note that for a Poisson distribution, the mean is equal to the variance. We test this expectation with a one-sample Kolmogorov-Smirnoff (KS) test. *Z* is the KS test statistic, and *P* its significance. Not a single test remained significant after applying the conservative Holm-Bonferroni correction for multiple tests of the same null hypothesis. The variance appears to deviate more from the mean at high doses, however. N.D. indicates data were not determined for a particular replicate and dose.

**Table 2:** Variance in the mean number of foci of primary infection for *C. annuum*.

Dilution	Virion Dose	Replicate	Mean foci	Variance	*Z*	*P*
1∶10	6.91×10^7^	1	2.80	7.60	0.654	0.785
		2	7.60	22.97	0.656	0.783
		3	5.33	6.67	0.680	0.744
1∶30	2.30×10^7^	1	2.13	1.98	0.359	1
		2	1.20	0.74	0.501	0.963
		3	2.80	6.46	0.797	0.549
1∶90	7.68×10^6^	1	0.93	0.50	0.490	0.970
		2	0.14	0.13	0.036	1
		3	0.21	0.18	0.080	1
1∶270	2.56×10^6^	1	0.47	0.55	0.206	1
		2	0.33	0.38	0.085	1
		3	0.07	0.07	0.008	1
1∶810	8.53×10^5^	1	N.D.			
		2	0.07	0.07	0.008	1
		3	0	0	-	-

Dilution is the dilution of the purified virions used to inoculate plants, and virion dose is the number of virions per inoculated plant, as estimated by the number of RNA molecules measured by RT-qPCR. Mean foci is the mean number of foci of primary infection, of both TEV-GFP and TEV-mCherry, in an inoculated leaf. Variance is the calculated variance of the mean number of foci of primary infection. Note that for a Poisson distribution, the mean is equal to the variance. We test this expectation with a one-sample Kolmogorov-Smirnoff (KS) test. *Z* is the KS test statistic, and *P* its significance. Not a single test remained significant after applying the conservative Holm-Bonferroni correction for multiple tests of the same null hypothesis. N.D. indicates data were not determined for a particular replicate and dose.

**Figure 3:**
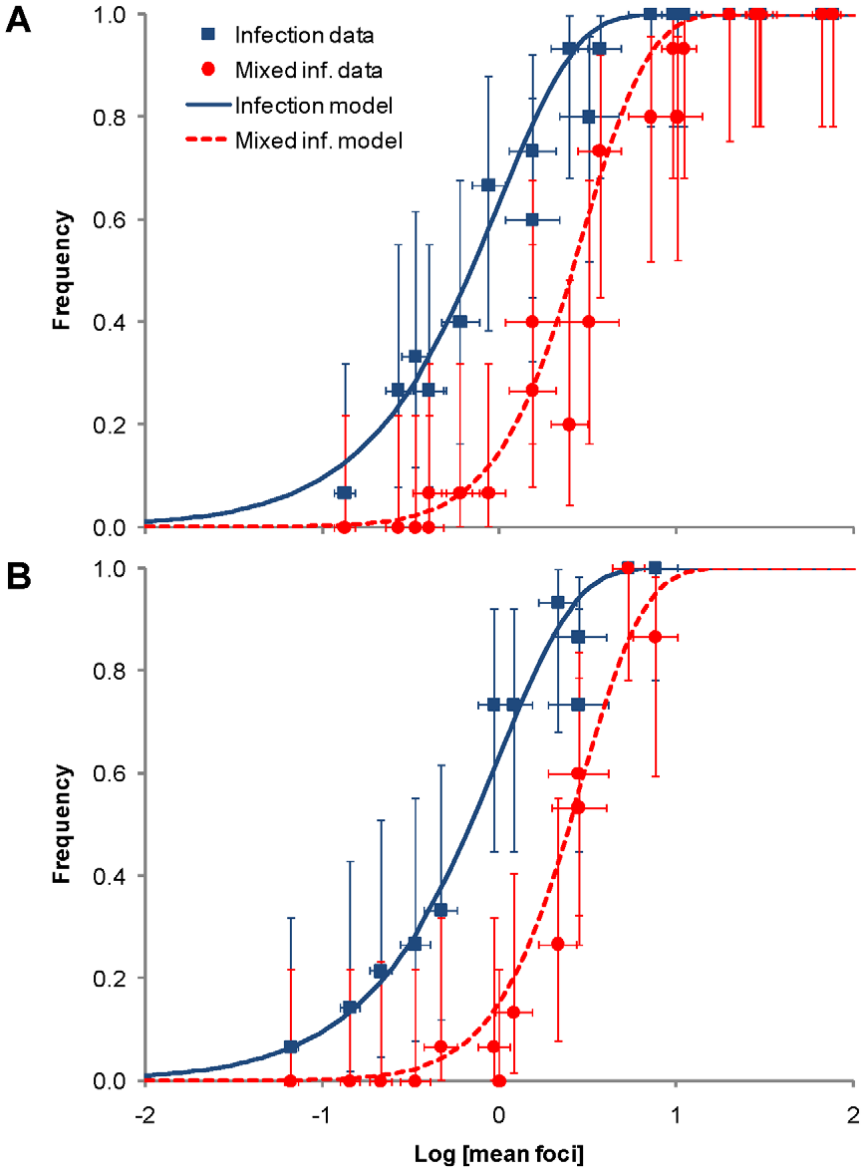
The rate of infection and mixed-genotype infection predicted from the number of primary infection foci in the inoculated leaf.

If the number of foci in inoculated leaves follows a Poisson distribution, then we should also be able to predict the zero term from the mean [7], [17]. As we expect single focus of primary infection to lead to systemic infection, we predicted the rate of systemic infection (*I*), for each experimental replicate, from *λ*:
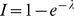
(1)Systemic infection status of a plant was determined by checking the plants for green or red fluorescence in non-inoculated leaves at 180 hpi. The data are in good agreement with the model predictions for both *N. tabacum* and *C. annuum* (Fig. 3; see also Tables S1 and S2 in Text S1), providing further evidence that the number of founders does indeed follow a Poisson distribution.

### Dose-response curves are compatible with IAH predictions

We considered the relationship between the number of foci at the inoculated leaf and response (i.e., systemic infection). Dose response has, however, often been used as a test for IAH [14], [17], [39], [40], [41], [42] because the IAH model with a fixed probability of infection predicts a dose response with a fixed shape [42]. We therefore fit a general dose-response model [42] to the data from each experimental replicate (see Materials and Methods). Table 3 shows the estimated parameters as well as the results of the one-sample *t*-test evaluating whether the estimates of the *κ* parameter significantly deviate from the IAH expectation of *κ* = 1. In the case of *N. tabacum*, three estimates were smaller than one, although the difference was significant only in one case. Treating each estimate of *κ* as a replicate, we can test whether an overall trend exist to deviate from the IAH expectation. The average estimate of *κ* (± SEM) for *N. tabacum* was 0.931±0.144, a value that is not significantly different from one (*t*_3_ = 0.479, *P* = 0.663). In the case of *C. annuum*, *κ* was significantly larger for the third replicate (Table 3). The average estimate of *κ* for *C. annuum* experiments was 1.215±0.156, a value that does not deviate from the IAH expectation (*t*_2_ = 1.378, *P* = 0.302). Therefore, although heterogeneity among replicates may exist, on average, dose response is also in agreement with IAH predictions. This result is not surprising given that there is a linear relationship between dose and number of foci, and that the foci response was also similar to IAH predictions. Indeed, results are qualitatively the same if instead the number of foci is used as doses (data not shown).

**Table 3:** Parameters estimated for the fitting of the dose response general infection model to each dataset.

Host	Replicate	*ρ* (±1 SEM)	*κ* (±1 SEM)	*P*
*N. tabacum*	1	(3.12±1.68)×10^−6^	0.685±0.129	0.072
	2	(2.37±0.87)×10^−6^	0.816±0.100	0.124
	3	(0.93±1.17)×10^−6^	1.346±0.396	0.422
	4	(6.89±0.29)×10^−6^	0.876±0.016	0.001^*^
*C. annuum*	1	(0.43±1.01)×10^−7^	0.973±0.356	0.994
	2	(0.39±1.24)×10^−8^	1.166±0.466	0.740
	3	(3.75±1.67)×10^−10^	1.506±0.064	0.001^*^

*P* values are the significance level of the corresponding one-sample *t*-test for the null hypothesis *κ* = 1. Asterisks indicate cases that remain significant after the more stringent Holm-Bonferroni correction.

### Foci with mixed TEV-GFP and TEV-mCherry infections occur at low frequency

**Figure 4:**
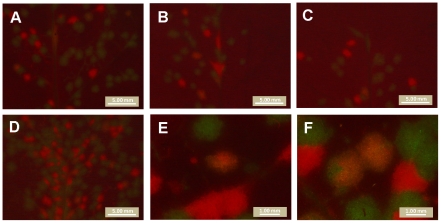
Mixed-genotype foci of primary infection in the inoculated leaf. Plants were infected with 1∶30 dilution of a mixture of amplified TEV-GFP and TEV-mCherry virions, kept on ice for 0 h (panel A), 2 h (panel B) or 3 h (panel C). Yellow, mixed-genotype foci were observed at low frequency at all three time points (1.8% averaged for all three times), and the frequency of mixed-genotype foci did not change significantly over time (test for a trend in proportions: χ^2^ = 0.041, 1 d.f., *P* = 0.839). Plants were also infected with a 1∶10 dilution of the same mixture (Panel D): the number of mixed-genotype foci may be higher, but these leaves could not be quantitatively scored. High magnification pictures of yellow foci are also provided (Panels E and F). Note that the distribution of red and green fluorescence is not homogeneous within yellow foci.

Mixed TEV-GFP and TEV-mCherry foci of primary infection were not observed in the inoculated leaves when counting the number of foci. However, we wanted to ascertain whether multiple virions could in principle be responsible for inducing a single primary infection focus, because virions of plant viruses are known to aggregate [43]. We therefore amplified TEV-GFP and TEV-mCherry to obtain virus stocks with higher virion concentrations, and inoculated *N. tabacum* plants with a 1∶30 dilution of a 1∶1 mixture of these viruses (2.30×10^7^ RNA molecules, as measured by RT-qPCR). Yellow primary infection foci – indicating mixed-genotype infection – were observed at a low frequency (Fig. 4). The frequency of mixed-genotype foci was estimated at 1.8% for this dose. The number of mixed-genotype foci may be greater at higher doses (1∶10 dilution of virions), but the number of foci could no longer be quantified (Fig. 4D). The same experiment was also performed on virions obtained from mixed-genotype infected plants, or virions extracted from a 1∶1 mixture of single-genotype infected tissues, with similar results and no differences in the frequency of mixed foci between the different treatments (data not shown). Our results have some similarities to those recently reported for *Soil-borne wheat mosaic virus* (SBWMV) [44], although this study explored co-infection at the cellular level. This work describes high levels of cellular co-infection upon saturation of the leaf with RNA, followed by lower levels of co-infection after each round of viral spread and new cellular infection. Similarly, we found much lower levels of co-infection in primary foci of infection, and we did not observe any co-infections in subsequently infected tissues, be it in the inoculated or systemically infected leaves.

Our data show that multiple virions can in principle initiate a single locus of primary infection. On the other hand, the frequency at which these mixed-infections occur appears to be very low at the doses tested here. Most foci of primary infection are therefore probably initiated by a single virion, and we therefore conclude that the number of primary infection foci is, at all but extremely high doses (>100 foci per leaf), a very good approximation of the number of virions infecting the inoculated leaf. The number of virions infecting the inoculated leaf is, consequently, also dose dependent.

### A mechanism of dependence between virions in systemic infection

**Figure 5:**
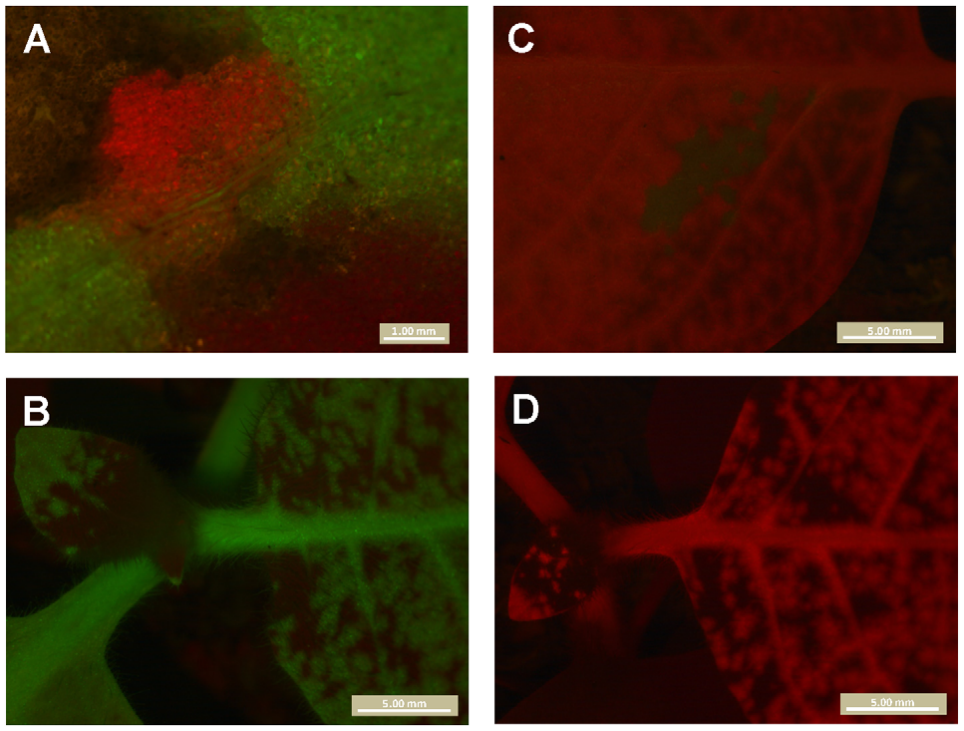
A mechanism of dependence between virions during infection of *N. tabacum*. All panels are merges of red and green fluorescence observed in *N. tabacum* plants inoculated with TEV-GFP and TEV-mCherry. Panel A is the only TEV-mCherry primary infection focus observed in the inoculated leaf, which at 132 hpi is surrounded by TEV-GFP and tissue damaged by the inoculation. Note that vascular tissue is present directly below the focus, but that only the TEV-GFP virus is visible there. In B, a view of systemically infected leaves is shown. No red fluorescence was seen in systemically infected leaves, indicating TEV-mCherry was trapped in the primary infection focus. In Panel C is the only green fluorescence observed in another plant. Although the TEV-GFP virus appears to have spread locally in the inoculated leaf, it is entirely surrounded by TEV-mCherry, and no green fluorescence was visible in systemically infected leaves (Panel D). Although these observations represent a mechanism of dependence between virions in the infection process (antagonism), ‘trapping’ of one of the two viruses in the inoculated leaf was observed only in 1.8% of the infected *N. tabacum* plants.

**Figure 6:**
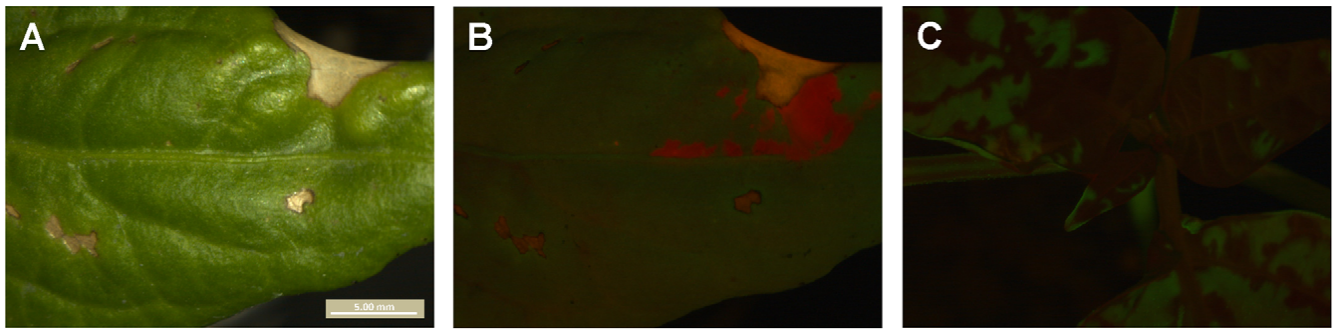
Dependence of virions during infection of *C. annuum*. In panel A, the inoculated leaf of a *C. annuum* plant is shown. Note the areas of the leaf damaged due to inoculation. In panel B, the same region of the leaf is shown but with merged GFP and mCherry signal. mCherry is restricted to small portion of the leaf, probably also in part due to leaf damage from inoculation. On day three, there were three mCherry foci visible in the inoculated leaf. Note that the yellow signal in this panel is due to leaf damage, and not co-infection of tissue. In panel C, the rest of the plant is shown, and only GFP signal is present. TEV-mCherry has therefore been ‘trapped’ in the inoculated leaf. This exclusion was observed in only one infected *C. annuum* plant (0.9%).

We determined which virus genotypes had systemically infected the plant by checking for red and green fluorescence in all leaves other than the inoculated leaf at 180 hpi. When red or green fluorescent foci of primary infection were observed in the inoculated leaf, the respective virus was almost always found systemically. The probability of establishing systemic infection after primary infection is therefore 1, or nearly 1, as demonstrated by Fig. 3. Including a pilot experiment, a total of 523 *N. tabacum* plants were inoculated, and 275 became infected. Of these 275 infected plants, there were only 3 plants in which a genotype was present in the primary infection foci, but not in the systemically infected leaves. In all three cases, the single locus of primary infection in the inoculated leaf was completely surrounded by the other genotype, probably cutting off access to the vascular tissue and preventing further spread of the virus in the inoculated leaf and systemically (Fig. 5). 265 *C. annuum* plants were inoculated in total, and 114 became infected; of these, there was only 1 plant in which a genotype was present in the primary infection foci, but not in the systemically infected leaves. However, in this one case there were three GFP foci of primary infection in the inoculated leaf, which were completely surrounded by the other genotype (Fig. 6). The host does not have any significant effect on the frequency at which foci are surrounded and precluded to move systemically among host (Fisher's exact test *P* = 0.664).

Interference between genotypes has been described for super-infection of different strains or co-inoculation of different plant viruses [24], but to the best of our knowledge not for the synchronous co-infection of multiple genotypes. Although we have identified potential mechanism of dependence between virions, the low frequency (1.8% of infected *N. tabacum* plants, and 0.9% of infected *C. annuum* plants) at which one genotype blocked systemic infection by the other genotype make it negligible in this experimental setup. However, when a large number of genotypes are present in the viral inoculum, it becomes more likely that one or more genotypes may be blocked from systemic infection. The same holds for *de novo* genetic variation arising early in the infection process.

### Frequency of mixed-genotype infections is predicted by an independent-action model

Although the number of primary infection foci appears to be dose-dependent (Fig. 2), we also sought to test whether the *N_e_* for the whole infection process is also dose-dependent. We used the frequency of mixed-genotype infection in systemically infected leaves as an indicator for population size in systemic infection. If both genotypes had systemically infected the plant, then both genotypes were usually present in all infected leaves as well. We predicted the frequency of mixed-genotype systemic infection based on the mean number of primary infection foci (*λ*) per replicate. We partitioned *λ* into *λ*_R_ and *λ*_G_, the number of infecting virions for TEV-mCherry and TEV-GFP, based on the proportion of foci of each genotype at the 1∶10 virion dilution. Following Zwart *et al.* [7], we estimated the frequency of mixed-genotype systemic infection in all hosts *P*(R ∩ G), both infected and non-infected, as:
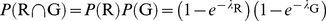
(2)Note that this model again assumes that each focus of primary infection will lead to systemic infection of the plant. The data corresponded well to model predictions (Fig. 3; see also Tables S1 and S2 in Text S1). The IAH model of infection, with a fixed probability of infection, is therefore well supported by the mixed-genotype systemic infection data.

The high probability of systemic infection following primary infection, and the fact that the mean number of primary infection foci (Fig. 3) predicted the frequency of mixed-genotype infection, support the hypothesis that *N_e_* at the level of systemic infection is also dose-dependent. The probability of systemic infection following primary infection is approximately 1, and the size of *N_e_* for systemic infection is therefore very similar to *N_e_* in the inoculated leaf (Fig. 2). As an estimate of *N_e_* at different doses, we therefore use the mean number of foci of primary infection in infected plants (Table 4). The similarity of *N_e_* in the inoculated and systemically infected leaves contrasts with previous work, in which there is a bottleneck between primary and systemic infection [28], [29], [30], [31], [33], and presumably a low probability of causing systemic infection after having caused primary infection (summarized in Table 4). This difference is undoubtedly associated to plant immune defenses, although the underlying mechanism is unclear. One way to approach this question would be to consider whether the probability of causing systemic infection is independent for each virion in these pathosystems. If there is independence between infecting virions, non-adaptive – and probably local – defense mechanisms in the leaf would appear to be responsible. If there is dependence between infecting virions, an adaptive immune defense – perhaps acting at the systemic level – would be implicated.

**Table 4:** Comparison of estimates of *N_e_*.

Ref.	Setup	Virus	Host	Dose	Infection	*N_e_*
TS	II,III; virions of 2 variants with fluorescence markers	TEV	Tobacco	1.73×10^7^	1.00	47.86±3.26
TS	II,III; virions of 2 variants with fluorescence markers	TEV	Tobacco	5.75×10^6^	1.00	18.63±2.29
TS	II,III; virions of 2 variants with fluorescence markers	TEV	Tobacco	1.92×10^6^	0.88	6.66±0.82
TS	II,III; virions of 2 variants with fluorescence markers	TEV	Tobacco	6.39×10^5^	0.72	2.66±0.63
TS	II,III; virions of 2 variants with fluorescence markers	TEV	Tobacco	2.13×10^5^	0.38	1.20±0.55
TS	II,III; virions of 2 variants with fluorescence markers	TEV	Tobacco	7.10×10^4^	0.22	1.40±0.32
TS	II,III; virions of 2 variants with fluorescence markers	TEV	Pepper	6.91×10^7^	0.96	5.39±2.19
TS	II,III; virions of 2 variants with fluorescence markers	TEV	Pepper	2.30×10^7^	0.80	2.58±1.12
TS	II,III; virions of 2 variants with fluorescence markers	TEV	Pepper	7.68×10^6^	0.36	1.09±0.16
TS	II,III; virions of 2 variants with fluorescence markers	TEV	Pepper	2.56×10^6^	0.22	1.22±0.20
TS	II,III; virions of 2 variants with fluorescence markers	TEV	Pepper	8.53×10^5^	0.03	1.00
[28], [29]	III; sap of 2 natural variants with RE markers	WSMV	Wheat	N.D.	N.D.	3–5
[30]	II; virions of 2 variants distinguishable by dot blot^a^	TMV	Tobacco	300 ng	0.96	3.1–5.6^*^
[30]	III (systemic leaf 1); virions of 2 variants distinguishable by dot blot^a^	TMV	Tobacco	300 ng	0.96	1.3–2.4^*^
[30]	III (systemic leaf 2); virions of 2 variants distinguishable by dot blot^a^	TMV	Tobacco	300 ng	0.96	2.6–4.2^*^
[31], [32]	III; RNA of 12 variants with RE markers	CMV	Tobacco	-	1.00	12–20
[33]	III; RNA of 6 variants with marker sequences	CaMV	Mustard	-	1.00	298–484
[25]	I; 2 variants distinguishable by ELISA, vector *Myzus persicae*	PVY	Pepper	-	0.29^b^	0.5–3.2^*,^ c
[26]	I; 12 variants with RE markers, vector *Aphis gossypii*	CMV	Squash	-	1.00	2.94±0.17^d^
[26]	I; 12 variants with RE markers, vector *M. persicae*	CMV	Squash	-	1.00	2.84±0.15^d^
[27]	I; 2 variants distinguishable by dot blot^e^, vector: *A. gossypii*	CMV	Tomato	-	0.23	1.19–1.73^*^
[27]	I; 2 variants distinguishable by dot blot^f^, vector: *A. gossypii*	CMV	Tomato	-	0.14	1.17–1.98^*^

TS stands for ‘this study’. RE stands for restriction enzyme. The experimental setups were classified according to which part of the transmission or infection process was considered for *N_e_* was estimated: I. Full transmission process: systemic leaf – vector – inoculated leaf – systemic leaf. II. Partial infection process: inoculum - inoculated leaf. III. Partial infection process: inoculated leaf – systemic leaf. Doses are given as the number of RNA molecules measured in purified virions stocks for data from this study, or as ng of purified virions per inoculated plant for study [30]. *N_e_* estimates are given with the standard error of the mean, or the reported range of estimates. Estimates of *N_e_* are made for (systemically) infected hosts, with the exception of those estimates marked with an asterisk (*) which are estimates made over the whole host population, not only the infected hosts.

a Only data from the wild-type/P20L experiment presented, as other experiments with other genotype combinations gave highly similar results.

b *N_e_* estimates made for different mixtures of an infective and non-infective genotype used, leading to different levels of infection. The mean level of infection is given.

c The estimated number of virions transmitted per aphid; 2 aphids per plant were used in the study.

d The mean number of genotypes found in infected plants is given, which is a rough approximation of *N_e_* as the number of genotypes used (12) is much higher than the number of genotypes found in infected plants.

e F1F2F3 and F1L2F3 genotypes.

f F1F2F3 and F1L2F3 genotypes, and satellite.

### Concluding remarks

We provide strong evidence from different experimental approaches that *N_e_* is dose-dependent for TEV, a plant RNA virus. Moreover, the relationship between *N_e_* and dose can be described with a simple mathematical model. This means that comparison of estimates of *N_e_* for different plant viruses (Table 4) is incomplete without taking into account dose effects as discrepancies between estimates of *N_e_* at a single dose may be due in part to dose-dependence. In previous work estimating *N_e_* for systemic colonization, most or all plants were infected (Table 4), meaning that the actual doses used to achieve these high levels of systemic infection could therefore have varied widely, despite causing high levels of systemic infection in all cases. Note that all other estimates of *N_e_* fall within the range of estimates we made (approx. 1 to 48, see Table 4), with the exception of the dsDNA pararetrovirus CaMV [33]. We speculate that the independent-action model may very well apply to many mono-partite plant viruses, but this will require experiments considering the frequency of mixed-genotype infection at different doses, as we have done here.

Dose-dependent *N_e_* was observed in host plants with a high (*N. tabacum*) and low susceptibility (*C. annuum*). Therefore, the only appreciable difference between TEV infections of these two plant species was the probability that a virion causes a focus of primary infection in the inoculated leaf. This probability was estimated to be ∼53 times lower for *C. annuum* than for *N. tabacum* when considering the number of foci in the inoculated leaf, and ∼52 times lower when considering mean values of *ρ*. It has been suggested the failure of the IAH may be linked to resistance of the host [7], while here we show that this does not, in principle, have to be the case. There are of course important differences between the infection process in these two hosts (i.e., note the differences in viral expansion in the inoculated leaf between Fig. 1 and Fig. S1), but basic infection parameters of the number of foci of primary infection and the frequency of mixed-genotype infections are adequately described by the IAH model.

At low doses, we regularly observed systemic infection caused by a single focus of primary infection. Although leaves were exposed to a moderate number of virions (∼10^5^ RNA molecules, see Table 4), only a very small number of foci, approaching 1, were observed. As our data also indicate that most foci of primary infection are initiated by a single virion, we conclude that systemic infection initiated by a single virion will regularly occur at low doses. Therefore, plant RNA virus *N_e_* can be extremely small and the impact of genetic drift on virus populations can be tremendous. The fact that *N_e_* is dose-dependent and can be very small is relevant for experimental evolution with plant viruses. If the typical dose of virions transmitted in the field by vectors also proves to be small, as shown for another plant RNA virus [25], [27], this will demonstrate the dominant role genetic drift plays in plant virus evolution and highlight the importance of mechanisms to buffer its effects [45].

## Materials and Methods

### Construction of TEV-mCherry and TEV-GFP

Plasmid pMTEV is a transcription vector containing a TEV infectious clone (Genbank accession DQ986288) [34] and was used as starting point to construct the TEV-GFP and TEV-mCherry genotypes in which these two fluorescent marker genes were inserted between TEV P1 and HC-Pro cistrons. GFP or mCherry cDNA [46] was amplified by PCR using primers forward 5′-ATGGTGAGCAAGGGCGAGGAGCTG-3′ and reverse 5′-TTGGAAGTACAAGTTTTCTCCGCCGAGGTCTGAGTACTTGTAC-3′ (GFP), or forward 5′-ATGGTTAGCAAAGGCGAGG-3′ and reverse 5′-TTGGAAGTACAAGTTTTCTCCGCCCTTATACAGCTCATCCATG-3′ (mCherry). The reverse primers inserted two glycines, as spacers, and a partial NIa-Pro proteolytic site downstream of GFP or mCherry sequences. This partial proteolytic site (ENLYFQ) is complemented by the first contiguous serine in HC-Pro cistron to mediate marker release from the viral polyprotein. The pTV1a vector, which contains the first 3221 nt of the TEV genome including the complete P1 to HC-Pro cistrons, was amplified using the forward primer 5′-AGCGACAAATCAATCTCTGAGGC-3′ and reverse primer 5′-TTTGTCGCTATAATGTGTCATTGAG-3′. The PCR-amplified GFP and mCherry sequences were then ligated into the amplified vector sequence, and transformed into electrocompetent *Escherichia coli* DH5α. The identity of the resulting pTV1a-GFP and pTV1a-mCherry plasmids was checked by restriction digests and sequencing. Finally, PauI-AatII restriction fragments from pTV1a-GFP and pTV1a-mCherry were ligated into PauI-AatII digested pMTEV to construct pTEV-GFP and pTEV-mCherry. All PCR reactions were performed with high fidelity Phusion DNA polymerase (Finnzymes).

### Reconstitution, purification and quantification of virions

We required virions of TEV-mCherry and TEV-GFP at equal concentrations in order to make stoichiometric virion mixes. To this end, RNA was transcribed from pMTEV-GFP and pMTEV-mCherry as described elsewhere [47]. Four-week-old *Nicotiana tabacum* var. Xanthi plants were inoculated with ca. 5 µg of RNA in the third true leaf, and systemically infected leaves harvested 9 days post inoculation (dpi). Virions were extracted from leaves as described elsewhere [47], although half the amount of infected tissue was used as starting material, and all volumes in the procedure halved. Virions were resuspended in 100 µL 0.05 M borate buffer (pH 8.0, 5 mM EDTA) with 20% glycerol, and stored as 15 µL aliquots.

In order to quantify virion concentration, we used a RT-qPCR assay. The assay was similar to that described by Carrasco *et al.* [47], with a number of modifications. 5×10^5^ copies of *in vitro* transcribed full-length *Turnip mosaic virus* (TuMV) genomic RNA were added to each sample at the start of RNA extraction, as an internal control. RNA was extracted using the RNeasy Plant kit (Qiagen), following the manufacturer's instructions. 10 µL of the purified virions was used as starting material for the extraction. RT was performed with the TaqMan kit (Applied Biosystems), using an oligo(dT)_16_ primer. qPCR amplification was performed separately for TEV (forward primer: 5′-TTGGTCTTGATGGCAACGTG-3′ and reverse primer 5′-TGTGCCGTTCAGTGTCTTCCT-3′) and TuMV (5′-GGCACTCAAGAAAGGCAAGG-3′ and 5′-TTGTCGCGTTTTCCCTCTTC-3′). 20 µL total reaction volume was used, with 10 µL 2× SYBR Green PCR Master Mix (Applied Biosystems), 2 µL cDNA template and 300 nM of each primer. RT and qPCR were performed in triplicate for each sample. For the standard curve, serially diluted quantified DNA of pMTEV and p35Tunos [48] plasmids were used. An ABI PRISM Sequence Analyzer 7000 was used to amplify the DNA with the following thermal profile: 10 min at 95°C; 40 cycles of 30 s at 95°C and 1 min at 60°C. Concentrations of TEV and TuMV cDNA were calculated with SDS7000 software version 1.2.3 (Applied Biosystems), and TuMV concentrations were used to normalize TEV concentrations. We did not find appreciable differences in concentration between virions simultaneously extracted, and therefore TEV-GFP and TEV-mCherry virions were mixed 1∶1 for inoculation experiments.

TEV-GFP and TEV-mCherry were amplified in 3-week-old *N. tabacum* plants, following inoculation of a 1∶10 dilution of sap by abrasion. Viruses were amplified separately, or by a 1∶1 mixture of virions. For 1∶1 mixtures, plants that showed equal levels of systemic infection for both viruses, and the highest levels of mixing of fluorescence in the leaves were selected (‘mixed-infected plant virions’). Systemically infected leaves were harvested 9 dpi, and virions purified as described above. We also purified virions from a 1∶1 mixture (by weight) of tissues (‘mixed-tissue virions’). RNA extractions and RT-qPCR were performed, and no appreciable differences were found between samples. TEV-GFP and TEV-mCherry virions were mixed 1∶1 (‘mixed virions’) treatment, and equivalent volumes of ‘mixed-infected plant’ and ‘mixed-tissue’ virions used for experiments. Note that the concentration of these virions was higher than those originating from plants infected with RNA.

### Inoculation experiments with mixtures of TEV-GFP and TEV-mCherry

*N. tabacum* plants that were 25 days old were used for inoculation experiments, and a selection of plants to be included in the experiment was made based on size. Plants were inoculated by abrasion of the third true leaf with a mixture of virions, with dilutions made in the 0.05 M borate buffer. Five µL of virion dilution were added to each leaf. Plants were subsequently kept in a growth chamber at 24°C. Fluorescence was observed with a Leica MZ16F stereomicroscope, using a 0.5× objective lens, and GFP2 and DSR filters (Leica) to view GFP and mCherry, respectively.

For a pilot experiment in *N. tabacum*, 15 plants were inoculated with the following virion dilutions: 1∶10, 1∶30, 1∶90, 1∶270, 1∶810, 1∶2430, 1∶7290 and a non-virus control. No infections were observed with a 1∶2430 and 1∶7290 dilutions of the pilot experiment. The number of TEV-GFP foci was ca. two-fold higher than the number of TEV-mCherry foci in the pilot experiment, so the dose of TEV-mCherry was doubled for the definitive experiment. For the definitive experiment, four replicates were performed. For replicate 1, 15 plants each were used with three-fold dilutions from 1∶10 until 1∶810, and a non-virus control. For replicates 2 to 4, 15 plants each were used with three-fold dilutions from 1∶10 until 1∶2430, and a non-virus control.

For experiments in *Capsicum annuum* var. Marconi, 32 day old plants were selected based on size. TEV-GFP and TEV-mCherry virions that had undergone one round of amplification in *N. tabacum* (i.e. ‘mixed virions’) were used for the inoculum, again with double the dose of TEV-mCherry. Inoculation and experimental conditions used were otherwise identical to those for *N. tabacum*. For a pilot experiment, 12 plants were inoculated with a 1∶10 mixture. For replicate 1 of the definitive experiment, 15 plants were inoculated with the following virion dilutions: 1∶10, 1∶30, 1∶90, 1∶270, 1∶810, and a non-virus control. For replicates 2 and 3, a 1∶2430 dilution was also included.

### Testing reporter gene stability

To test the stability of the reporter genes, four-week-old *N. tabacum* plants were infected with TEV-mCherry or TEV-GFP virions by abrasion, with five replicates for each virus. One week post inoculation, infected tissues were collected and a 1∶10 dilution of sap was inoculated into new plants by abrasion. After one week, infected tissues were collected, and fluorescence was observed by stereomicroscope to be equivalent to that in plants infected with TEV-GFP or TEV-mCherry RNA (data not shown). RNA was extracted with the RNeasy plant kit (Qiagen), and RT was performed using M-MuLV (Fermentas) and random hexamers as a primer. A Taq polymerase (Roche) based PCR was performed with two sets of specific primers flanking the reporter gene (set 1, forward primer: 5′-CAATTGTTCGCAAGTGTGC-3′, reverse primer: 5′-ATGGTATGAAGAATGCCTC-3′; set 2, forward primer: 5′- GCAATCAAGCATTCTACTTC-3′, reverse primer: 5′- CCTGATATGTTTCCTGATAAC-3′). PCR products were resolved on a 1% agarose gel, and only the amplicon corresponding to TEV-GFP or TEV-mCherry was observed for the passaged viruses (data not shown). To test the sensitivity of the assay for genomes with the reporter gene deleted, we mixed TEV and TEV-mCherry RNA in different ratios. Primer set 1 could detect the small amplicon corresponding to TEV in at a ratio 1∶10,000, and primer set 2 could detect the TEV-derived amplicon at 1∶100.

### Statistical analysis

All statistical analysis was performed in SPSS version 16.0 (SPSS Inc.) unless otherwise noted.

To analyze the dose foci relationship, the foci data *λ* were transformed as *y* = ln(*λ*+1) and the relationship *y* = ln(*ϕd*+1) fitted using non-linear regression, where *ϕ* is the probability that a unit dose results in a focus of primary infection. One was added to *λ* because some leaves had zero foci; *d* stands for the dose.

The general model of dose-response relationships [42] is given by:
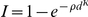
where *I* is the fraction of systemically infected plants, *d* is the virion dose as estimated by RT-qPCR, *ρ* is the average infection probability and *κ* determines what the effect of dose on the rate of infection is. When *κ* = 1, the dose-response relationship corresponds to the IAH. When *κ*>1, there is synergism between virions (the dose-response is steeper than predicted for the IAH), whilst when *κ*<1, there is antagonism (the dose-response is shallower than expected for the IAH). This model was fitted to the data using non-linear regression to estimate *ρ* and *κ*. For dose in *N. tabacum*, we divided the dilution by a constant, chosen for convenience (10^−4^). This constant is arbitrary because we are not interested in valid estimates of *ρ*, but rather we want an indication of the fit of the model. However, we wanted to compare these results to those in *C. annuum*. The number of foci of primary infection in the inoculated leaf of *N. tabacum* was therefore compared for the two virions stocks used for experiments in *N. tabacum* (virions purified from transfected *N. tabacum* plants) and *C. annuum* (virions purified from *N. tabacum* plants in the virus resulting from transfection was amplified). There were four-fold more foci generated by the amplified virion stock.

A two-tailed exact binomial test (R version 2.10.1, The R Foundation) was used to test whether the frequency of infections and mixed-genotype infections were significantly different from model predictions.

## Supporting Information

Figure S1 Fluorescence time course in inoculated *C. annuum* leaves. The development of foci of primary infection in the inoculated leaf was followed over time. Patterns in vascular tissue, observed under visible light through a stereomicroscope (‘Plant A: Vis.’), were used to track fluorescence in the same region. GFP and mCherry signals were merged, and given for three example plants (‘Plant A: Fluo.’ through ‘Plant C: Fluo.’). Note that only TEV-mCherry is present in Plant C, and that the tip of the leaf becomes necrotic over time. As for *N.* tabacum, the best time point for viewing foci of primary infection is 84 hpi; when the foci have reasonably high levels of fluorescence, but the fluorescence has not spread.
(TIF)

Text S1 Tables S1 and S2 comparing data and IAH model predictions. Table S1 compares data and model predictions for the rate of infection and mixed-genotype infection for *N. tabacum*. Table S2 makes the same comparisons for *C. annuum*.
(DOC)
