# Supplementary figures and images for: One Is Enough: In Vivo Effective Population Size Is Dose-Dependent for a Plant RNA Virus
Source: PLoS Pathog. 2011 Jul 7;7(7):e1002122. doi: 10.1371/journal.ppat.1002122 (PMC3131263; doi:10.1371/journal.ppat.1002122)

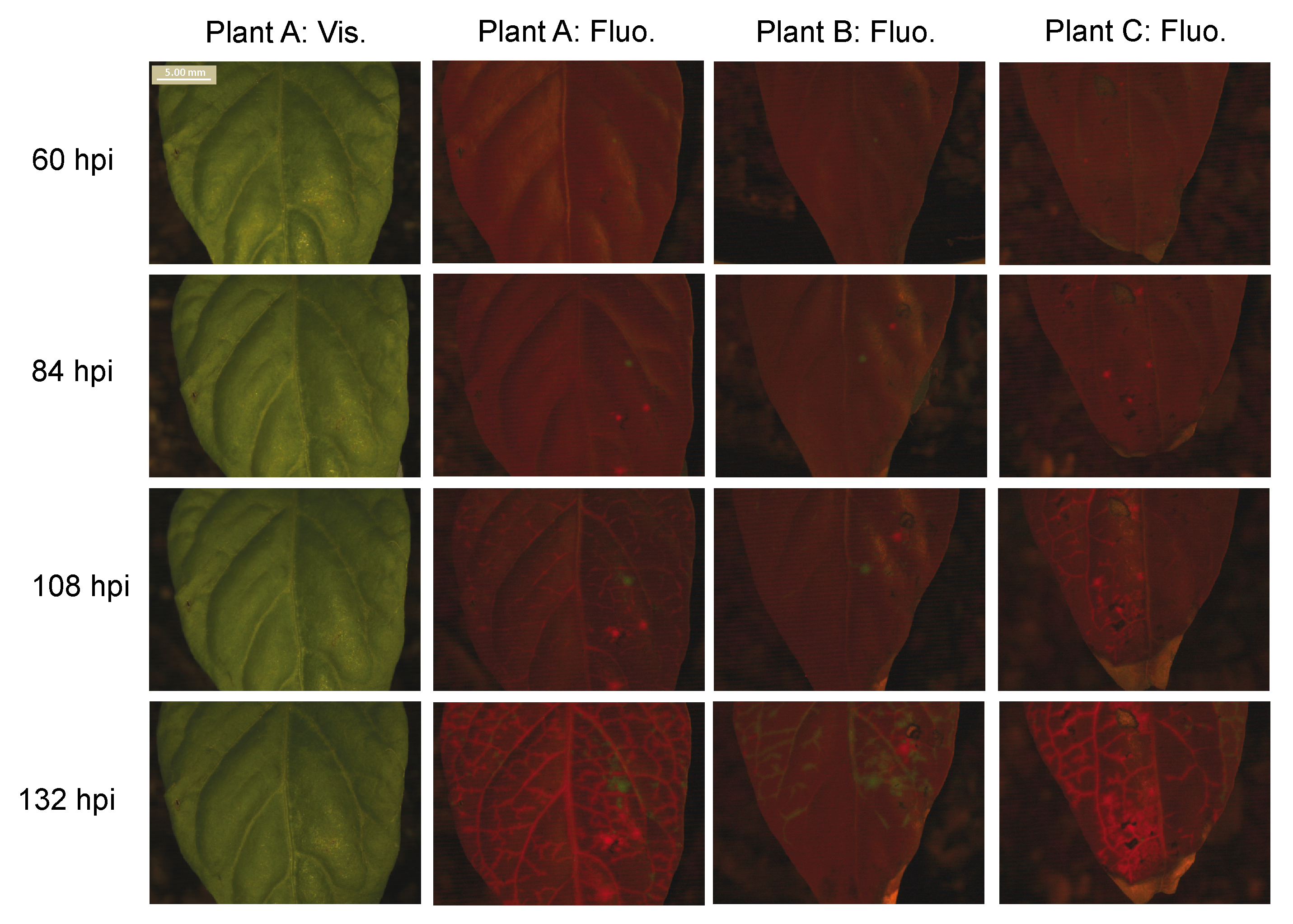

Supplement: Figure S1 — Fluorescence time course in inoculated C. annuum leaves. The development of foci of primary infection in the inoculated leaf was followed over time. Patterns in vascular tissue, observed under visible light through a stereomicroscope (‘Plant A: Vis.’), were used to track fluorescence in the same region. GFP and mCherry signals were merged, and given for three example plants (‘Plant A: Fluo.’ through ‘Plant C: Fluo.’). Note that only TEV-mCherry is present in Plant C, and that the tip of the leaf becomes necrotic over time. As for N. tabacum, the best time point for viewing foci of primary infection is 84 hpi; when the foci have reasonably high levels of fluorescence, but the fluorescence has not spread. (TIF) [file ppat.1002122.s001.tif]
