# Supplementary material for: One Is Enough: In Vivo Effective Population Size Is Dose-Dependent for a Plant RNA Virus
Source: PLoS Pathog. 2011 Jul 7;7(7):e1002122. doi: 10.1371/journal.ppat.1002122 (PMC3131263; doi:10.1371/journal.ppat.1002122)
Supplement: Text S1 — Tables S1 and S2 comparing data and IAH model predictions. Table S1 compares data and model predictions for the rate of infection and mixed-genotype infection for N. tabacum. Table S2 makes the same comparisons for C. annuum. (DOC) [file ppat.1002122.s002.doc]

**Table S1.** Comparison of data and model predictions for the rate of infection and mixed-genotype infection for *N. tabacum*.

|  |  |  |  | Dose foci relationship | | | Mixed-genotype infections | | |
| --- | --- | --- | --- | --- | --- | --- | --- | --- | --- |
| Dilution | Virion dose | Replicate | Mean foci | *I*_obs_ | *I*_pred_ | *P* | *P*_obs_(R ∩ G) | *P*_pred_(R ∩ G) | *P* |
| 1:10 | 1.73 × 10^7^ | 1 | 19.92 | 1 (13/13) | 1 | 1 | 1 (13/13) | 0.999 | 1 |
|  |  | 2 | 29.73 | 1 (15/15) | 1 | 1 | 1 (15/15) | 1 | 1 |
|  |  | 3 | 66.20 | 1 (15/15) | 1 | 1 | 1 (15/15) | 1 | 1 |
|  |  | 4 | 75.60 | 1 (15/15) | 1 | 1 | 1 (15/15) | 1 | 1 |
| 1:30 | 5.75 × 10^6^ | 1 | 7.13 | 1 (15/15) | 0.999 | 1 | 0.800 (12/15) | 0.922 | 0.107 |
|  |  | 2 | 9.00 | 1 (15/15) | 0.959 | 0.022 | 0.800 (12/15) | 0.976 | 0.005 |
|  |  | 3 | 28.00 | 1 (15/15) | 1 | 1 | 1 (15/15) | 1 | 1 |
|  |  | 4 | 29.33 | 1 (15/15) | 1 | 1 | 1 (15/15) | 1 | 1 |
| 1:90 | 1.92 × 10^6^ | 1 | 1.53 | 0.733 (11/15) | 0.784 | 0.546 | 0.267 (4/15) | 0.271 | 1 |
|  |  | 2 | 3.20 | 0.800 (12/15) | 0.784 | 0.110 | 0.400 (6/15) | 0.607 | 0.116 |
|  |  | 3 | 11.00 | 1 (15/15) | 1 | 1 | 0.933 (14/15) | 0.984 | 0.215 |
|  |  | 4 | 9.53 | 1 (15/15) | 1 | 1 | 0.933 (14/15) | 0.971 | 0.357 |
| 1:270 | 6.39 × 10^5^ | 1 | 0.60 | 0.400 (6/15) | 0.451 | 0.800 | 0.067 (1/15) | 0.063 | 1 |
|  |  | 2 | 1.53 | 0.600 (9/15) | 0.330 | 0.759 | 0.400 (6/15) | 0.271 | 0.256 |
|  |  | 3 | 3.67 | 0.933 (14/15) | 0.974 | 0.326 | 0.733 (11/15) | 0.674 | 0.786 |
|  |  | 4 | 2.47 | 0.933 (14/15) | 0.915 | 1 | 0.200 (3/15) | 0.477 | 0.038 |
| 1:810 | 2.13 × 10^5^ | 1 | 0.33 | 0.333 (5/15) | 0.283 | 0.775 | 0 (0/15) | 0.022 | 1 |
|  |  | 2 | 0.40 | 0.267 (4/15) | 0.330 | 0.786 | 0.067 (1/15) | 0.031 | 0.377 |
|  |  | 3 | 0.27 | 0.267 (4/15) | 0.234 | 0.762 | 0 (0/15) | 0.015 | 1 |
|  |  | 4 | 0.87 | 0.667 (10/15) | 0.580 | 0.606 | 0.067 (1/15) | 0.117 | 1 |
| 1:2430 | 7.10 × 10^4^ | 1 | N.D. |  |  |  |  |  |  |
|  |  | 2 | 0.13 | 0.067 (1/15) | 0.125 | 1 | 0 (0/15) | 0.004 | 1 |
|  |  | 3 | 0.40 | 0.267 (4/15) | 0.330 | 0.789 | 0 (0/15) | 0.031 | 1 |
|  |  | 4 | 0.33 | 0.333 (5/15) | 0.283 | 0.775 | 0 (0/15) | 0.022 | 1 |

Dilution is the dilution of the virus stock used to inoculate plants, and virion dose is the number of virions per inoculated plant estimated by RT-qPCR. The observed rate of infection (*I*_obs_) and mixed-genotype infection (*P*_obs_(R ∩ G)) are compared to model predictions (*I*_pred_ and *P*_pred_(R ∩ G), respectively). *P* is the significance value of binomial test comparing data and model predictions. Not a single *P* value remained significant after applying the conservative Holm-Bonferroni correction for multiple tests of the same null hypothesis. N.D. indicates data were not determined for a particular replicate and dose.

**Table S2.** Comparison of data and model predictions for the rate of infection and mixed-genotype infection for *C. annuum*.

|  |  |  |  | Dose foci relationship | | | Mixed-genotype infections | | |
| --- | --- | --- | --- | --- | --- | --- | --- | --- | --- |
| Dilution | Virion dose | Replicate | Mean foci | *I*_obs_ | *I*_pred_ | *P* | *P*_obs_(R ∩ G) | *P*_pred_(R ∩ G) | *P* |
| 1:10 | 6.91 × 10^7^ | 1 | 2.80 | 0. 867 (13/15) | 0.939 | 0.232 | 0.533 (8/15) | 0.558 | 1 |
|  |  | 2 | 7.60 | 1 (15/15) | 0.999 | 1 | 0.867 (13/15) | 0.949 | 0.176 |
|  |  | 3 | 5.33 | 1 (15/15) | 0.995 | 1 | 1 (15/15) | 0.856 | 0.150 |
| 1:30 | 2.30 × 10^7^ | 1 | 2.13 | 0.933 (14/15) | 0.882 | 1 | 0.267 (4/15) | 0.422 | 0.202 |
|  |  | 2 | 1.20 | 0.733 (11/15) | 0.669 | 1 | 0.133 (2/15) | 0.200 | 0.750 |
|  |  | 3 | 2.80 | 0.733 (11/15) | 0.939 | 0.010 | 0.600 (9/15) | 0.558 | 0.801 |
| 1:90 | 7.68 × 10^6^ | 1 | 0.93 | 0.733 (11/15) | 0.607 | 0.431 | 0.067 (1/15) | 0.136 | 0.710 |
|  |  | 2 | 0.14 | 0.143 (2/14) | 0.133 | 0.709 | 0 (0/14) | 0.005 | 1 |
|  |  | 3 | 0.21 | 0.214 (3/14) | 0.193 | 0.741 | 0 (0/14) | 0.010 | 1 |
| 1:270 | 2.56 × 10^6^ | 1 | 0.47 | 0.333 (5/15) | 0.373 | 1 | 0.067 (1/15) | 0.042 | 0.475 |
|  |  | 2 | 0.33 | 0.267 (4/15) | 0.283 | 1 | 0 (0/15) | 0.023 | 1 |
|  |  | 3 | 0.07 | 0.067 (1/15) | 0.064 | 1 | 0 (0/15) | 0.001 | 1 |
| 1:810 | 8.53 × 10^5^ | 1 | N.D. |  |  |  |  |  |  |
|  |  | 2 | 0.07 | 0.067 (1/15) | 0.065 | 1 | 0 (0/15) | 0.001 | 1 |
|  |  | 3 | 0 | 0 (0/15) | 0 | 1 | 0 (0/15) | 0 | 1 |

Dilution is the dilution of the virus stock used to inoculate plants, and virion dose is the number of virions per inoculated plant estimated by RT-qPCR. The observed rate of infection (*I*_obs_) and mixed-genotype infection (*P*_obs_(R ∩ G)) are compared to model predictions (*I*_pred_ and *P*_pred_(R ∩ G), respectively). *P* is the significance value of binomial test comparing data and model predictions for infection and mixed-genotype infection, respectively. Not a single *P* value remained significant after applying the conservative Holm-Bonferroni correction for multiple tests of the same null hypothesis. N.D. indicates data were not determined for a particular replicate and dose.
